# Correlates of intimate relationship satisfaction among investigators of child sexual abuse material

**DOI:** 10.3389/fpubh.2023.1237510

**Published:** 2023-11-07

**Authors:** Ateret Gewirtz-Meydan, Jennifer E. O'Brien, Kimberly J. Mitchell

**Affiliations:** ^1^School of Social Work, Faculty of Social Welfare & Health Sciences, University of Haifa, Haifa, Israel; ^2^Department of Social Work, University of New Hampshire, Durham, NH, United States; ^3^Crimes Against Children Research Center, University of New Hampshire, Durham, NH, United States

**Keywords:** child sexual abuse materials, investigators, forensic examiners, mental health, relationship satisfaction

## Abstract

**Introduction:**

This study investigates correlates of intimate relationship satisfaction among investigators of child sexual abuse material (CSAM). Previous research has shown that exposure to CSAM content can negatively impact investigators’ emotional wellbeing, but little is known about its association with their intimate relationships.

**Methods:**

The study included 500 participants who were police investigators, forensic examiners, and other professionals connected with the criminal justice system in the United States that are exposed to CSAM as part of their profession. The study collected data through an anonymous survey administered via the Qualtrics online survey system.

**Results:**

The findings reveal that higher levels of depressive symptoms and post-traumatic stress disorder are associated with lower relationship satisfaction among CSAM investigators. Additionally, group connectedness is positively related to relationship satisfaction for both men and women. Parent investigators also reported lower relationship satisfaction, suggesting unique challenges faced by this subgroup. Exposure to varying types of CSAM content was not significantly related to lower relationship satisfaction. This study highlights the association between depressive symptoms and PTSD with relationship satisfaction among CSAM investigators, emphasizing the role of group connectedness in promoting positive outcomes for both male and female investigators.

**Discussion:**

Recognizing the association between depressive symptoms and PTSD with relationship satisfaction can guide interventions and support services for investigators, promoting group connectedness and addressing mental health concerns to enhance resilience and effectiveness in combating child exploitation.

## Introduction

Child sexual abuse material (CSAM) has become a global concern, facilitated by advancements in online and digital technologies (1, 2). CSAM refers to sexually explicit depictions involving individuals under the age of 18, as defined by U.S. federal statutes (18 USCS 2256). The proliferation of the Internet since the mid-1990s has led to an increase in CSAM cases involving possession, distribution, and production. Specialized law enforcement units, like the 61 Internet Crimes Against Children (ICAC) Task Forces, typically handle such cases. These units consist of over 7,000 investigators and forensic examiners who may be exposed to explicit and graphic content while working on CSAM cases (3). In combating CSAM, investigators and forensic examiners have distinct but interconnected roles. Investigators conduct comprehensive investigations, including evidence collection, suspect identification, and legal proceedings, with a focus on apprehending offenders and protecting children. Forensic examiners, also known as digital forensic analysts, specialize in the technical aspects of CSAM investigations, extracting and analyzing digital evidence to support criminal prosecutions. During CSAM investigations, law enforcement personnel often encounter disturbing and explicit content graphically depicting child sexual abuse that not only transgress ethical norms but also portray distressing acts committed against child victims. The content of CSAM encountered by investigators can vary widely, encompassing images and videos depicting children of any gender and age (0–17 years), ranging from nudity to explicit sexual acts, and even the rape and torture of children (4).

Previous research has demonstrated significant emotional, cognitive, social, and behavioral consequences experienced by CSAM investigators following exposure to such material (4–7). Adverse outcomes include the development of posttraumatic stress symptoms (5, 8, 9), psychological distress (9), increased general distrust toward others (9), discomfort engaging in routine physical interactions with their own children (7), a sense of isolation from fellow law enforcement personnel (5), heightened vigilance to protect children and family (5, 9), as well as heightened sensitivity to child exploitation issues (5). Repeated exposure to CSAM is reported as a significant stressor for law enforcement professionals, which can lead to high burnout (5, 8, 10, 11). Investigators have also reported physical reactions such as headaches (5), mood swings (5), fatigue (5, 7), sleep disturbances (7), and emotional numbness (7). Law enforcement officers involved in CSAM cases also exhibit significantly higher levels of posttraumatic stress disorder (PTSD) compared to their non-CSAM-exposed counterparts, and experience symptoms such as intrusive imagery, hypervigilance, and mood fluctuations (5, 9, 12). Despite recognizing the adverse impact of CSAM exposure on the mental and physical health of investigators, there remains a notable scarcity of research investigating the intimate relationship satisfaction of individuals engaged in CSAM investigations.

Though intimate relationship satisfaction among CSAM investigators is notably absent from research, a few studies discuss the unique emotional toll of CSAM exposure. Specifically, in one qualitative study, CSAM investigators described “switching off” after work, and experiencing reduced interest in emotional and physical intimacy with their partners (7, 13). Such findings mirror research on other populations that have consistent exposure to trauma (e.g., military members, veterans, first responders, and healthcare workers) wherein PTSD and other mental health disorders have a negative impact on intimate relationship satisfaction (14–17). Similarly, the adverse effects of PTSD and mental health disorders on intimate relationships are also evident among survivors of sexual assault (18), and survivors of childhood maltreatment (19). Although the majority of studies examine how mental health and PTSD affect intimate relationships, this association can be bidirectional. Specifically, difficulties within intimate relationships can also contribute to or worsen existing mental health challenges (20). Given the similarities in trauma exposure and psychological consequences between investigators of CSAM and other populations that have experienced trauma, it is reasonable to hypothesize that mental health issues and PTSD may also be associated with the intimate relationship satisfaction of investigators exposed to CSAM.

Understanding the association between mental health (anxiety, depression and PTSD) and burnout on the intimate relationship satisfaction of investigators exposed to CSAM is an important step in facilitating wellbeing and effectiveness in their professional roles. The Spillover Model describes the process by which stressors (e.g., trauma exposure, workload, or burnout)—originating from external sources have the capacity to spill over into intimate relationships (21–23). Previous research has shown a significant interplay between work stress, mental health, and intimate relationships (24, 25). High levels of work-related stress have been negatively associated with intimate relationships, often leading to strained dynamics and decreased relationship satisfaction (26). High burnout (e.g., the demanding nature of work, prolonged working hours and heightened responsibilities), can leave individuals emotionally and physically drained (25), resulting in reduced availability and attentiveness toward their partners (27). Consequently, conflicts may arise more frequently, and couples may struggle to maintain effective communication and mutual support. Conversely, relationship difficulties can also contribute to work-related stress (24). When individuals experience unresolved conflicts or a sense of disconnection in their intimate relationships, their attention and emotional wellbeing may be compromised, making it challenging to concentrate on work-related tasks. The emotional toll of relationship problems adds an additional burden, leading to decreased productivity and reduced job satisfaction (24). In the context of the current study, the application of the Spillover Model can help elucidate how the challenges associated with managing the spillover of stress from CSAM investigation work may negatively impact the intimate relationship satisfaction of CSAM investigators. The cumulative impact of viewing traumatic and distressing material, such as CSAM can contribute to elevated levels of stress and emotional fatigue among professionals in this field (9–11, 28). Moreover, the complexities inherent in CSAM cases, including the need for meticulous attention to detail, the pursuit of justice, and the protection of vulnerable victims, can intensify the burden on these professionals (5, 8, 10). This heightened stress and emotional exhaustion can lead to a range of adverse consequences, including decreased job satisfaction, reduced effectiveness in investigations, compromised mental health, and perhaps also intimate relationships.

The Spillover Model highlights that stress at work can have a significant impact on personal relationships. However, it’s important to recognize that this relationship can work in both directions. Having strong support systems, including a sense of group connectedness and effective social support seeking, can contribute to interpersonal wellbeing, and reduced stress. Group connectedness and social support are concepts that can be examined from both within-group and out-group perspectives, each offering unique insights into how investigators seek and receive support in various contexts. Within-group connectedness refers to the sense of belonging, cohesion, and support experienced within a specific group or community to which an individual belongs (29, 30). This could include, for example, the connectedness that investigators working on CSAM cases feel within their specialized law enforcement units or task forces (31). Within-group connectedness involves the bonds formed among colleagues who share common goals, values, and experiences (29). In this context, investigators may turn to their fellow team members for emotional support, guidance, and understanding because they share the unique challenges and stresses of working on CSAM cases. On the other hand, out-group connectedness refers to seeking support or connections outside of one’s primary group or community. This might involve reaching out to individuals, organizations, or resources beyond the immediate circle of colleagues or team members (29). For investigators dealing with the emotional toll of CSAM cases, out-group connectedness could include seeking support from mental health professionals, employee assistance programs, or external peer support networks. However, it is important to note that the stigma surrounding mental health in military and police organizations, where seeking support is often viewed as a sign of weakness, significantly shapes perceptions of help-seeking and restricts access to professional support for many CSAM investigators (11, 31). Indeed, group connectedness and social support seeking have been shown to play vital roles in fostering resilience among police more broadly (32, 33). Being part of a supportive group provides a sense of solidarity, understanding, and shared identity (34, 35), which may alleviate the emotional toll of CSAM exposure. Seeking social support allows investigators to engage in open communication, gain access to resources, and receive validation and guidance. Both group connectedness and seeking social support can signify a greater sense of belonging and the desire for mutual connection, which enhances understanding and support within relationships, as they require trusting others, and involve actively seeking emotional support, which fosters a supportive environment. These factors contribute to effective communication, problem-solving, trust, intimacy, and stress reduction, all of which are associated with greater relationship satisfaction (36–38). It is unclear if these mechanisms work similarly among CSAM investigators.

### The current study

This study aims to address the research gap regarding intimate relationship satisfaction among CSAM investigators. Previous studies have shown that exposure to graphic CSAM content can be significantly related to lower emotional and psychological wellbeing of investigators (4–7). However, little is known about what is related to CSAM investigator’s satisfaction with their intimate relationships. By applying the Spillover Model (21, 22), this study aims to examine the association between mental health issues (namely anxiety and depression), PTSD, burnout, group connectedness, and seeking social support, and intimate relationship satisfaction among CSAM investigators. Drawing from research on other populations regularly exposed to trauma, it is hypothesized that mental health issues (namely anxiety and depression), PTSD, and burnout, will be negatively associated with the intimate relationship satisfaction of CSAM investigators. Conversely, we hypothesize that group connectedness and seeking social support will be positively related to intimate relationship satisfaction. Understanding these associations is crucial for their wellbeing and professional effectiveness, and can inform the development of support strategies and interventions. By fostering group connectedness and promoting social support seeking behaviors, organizations and support systems can enhance resilience and improve intimate relationship satisfaction among CSAM investigators.

## Methods

### Participants

Participants were 698 police investigators, forensic examiners, and others connected with the criminal justice system from the across the United States who were exposed to CSAM as part of their professions. The current paper included those participants who reported any CSAM exposure as part of their profession and had completed 85% of the survey questions, resulting in an analytic sample of 500 participants. Participants reported being exposed to CSAM nearly every day in a typical month (17.8%), 54.2% several days a month (54.2%), more than half the days (21%), or rarely (7%). Sixty-one percent of participants were male and 37.4% female; most were between the ages of 35–44 (39.8%) with an additional 21.6% aged 25–34 and 29.8% aged 45–54. The majority of participants was White race (85.8%) and 7.3% were of Hispanic or Latino ethnicity. Further details of the sample are depicted in Table 1.

**Table 1:** Demographic and job/agency characteristics.

Characteristic	All participants (*N* = 528) % (n)	Not in a relationship (*n* = 68) % (n)	In a relationship (*n* = 460)	*p-*value
Gender				
Male	59.7 (315)	35.3 (24)	63.3 (291)	<0.001
Female	39.2 (207)	61.8 (42)	35.9 (165)	
Non-binary	0.2 (1)	1.5 (1)	0	
Decline to answer	0.9 (5)	1.5 (1)	0.9 (4)	
Age				
18–24	1.0 (5)	4.5 (3)	0.4 (2)	0.03
25–34	22.4 (117)	26.9 (18)	21.8 (99)	
35–44	40.2 (210)	41.8 (28)	40.0 (182)	
45–54	29.7 (155)	22.4 (15)	30.8 (140)	
55–64	6.5 (34)	4.5 (3)	6.8 (31)	
65–74	0.2 (1)	0	0.2 (1)	
Hispanic ethnicity	7.5 (38)	4.8 (3)	7.9 (35)	0.40
Race^a^				
White	85.2 (450)	77.9 (53)	86.3 (397)	0.07
Black or African American	3.8 (20)	8.8 (6)	3.0 (14)	0.02
Asian or Pacific Islander	3.0 (16)	7.3 (5)	2.4 (11)	0.03
Native American or Alaska	1.3 (7)	2.9 (2)	1.1 (5)	0.21
Mixed racial background	3.0 (16)	1.5 (1)	3.3 (15)	0.42
Decline to answer	4.9 (26)	4.4 (3)	5.0 (23)	0.83
Marital status				
Married	68.9 (364)	1.5 (1)	78.9 (363)	<0.001
Unmarried but living with partner	6.1 (32)	0	7.0 (32)	
Separate or divorced	9.5 (48)	29.4 (20)	6.5 (30)	
Widowed	0.8 (4)	1.5 (1)	0.7 (3)	
Single (never married)	12.9 (68)	63.2 (43)	5.4 (25)	
Decline to answer	1.9 (10)	4.4 (3)	1.5 (7)	
Parent children under the age of 18 (any)	61.7 (326)	26.5 (18)	67.0 (308)	<0.001
Grandchildren under the age of 18 (any)	9.5 (50)	7.3 (5)	9.8 (45)	0.52
Number of years in current position				
Less than 1 year	8.9 (47)	10.3 (7)	8.7 (40)	0.25
2–3 years	32.6 (172)	33.8 (23)	32.4 (149)	
4–6 years	22.3 (118)	19.1 (13)	22.8 (105)	
7–10 years	15.0 (79)	13.2 (9)	15.2 (70)	
11–15 years	12.1 (64)	11.8 (8)	12.2 (56)	
16–20 years	5.9 (31)	11.8 (8)	5.0 (23)	
More than 20 years	3.2 (17)	0	3.7 (17)	
Number of years in field				
Less than 1 year	1.1 (6)	0	1.3 (6)	0.17
2–3 years	7.0 (37)	13.2 (9)	6.1 (28)	
4–6 years	11.5 (61)	11.8 (8)	11.5 (53)	
7–10 years	17.2 (91)	20.6 (14)	16.7 (77)	
11–15 years	22.2 (117)	25.0 (17)	21.7 (100)	
16–20 years	18.9 (100)	16.2 (11)	19.3 (89)	
More than 20 years	22.0 (116)	13.2 (9)	23.3 (107)	
Place of residence				
Large city (population over 300,000)	22.5 (118)	30.3 (20)	21.4 (98)	0.06
Smaller city (population about 100,000–300,000)	27.5 (144)	36.4 (24)	26.2 (120)	
Town (population about 20,000–100,000)	28.8 (151)	18.2 (12)	30.3 (139)	
Small town (population about 2,500–20,000)	16.8 (88)	10.6 (7)	17.7 (81)	
Rural area (population under 2,500)	4.4 (23)	4.5 (3)	4.4 (20)	
Type of agency work for				
Federal	12.5 (66)	20.6 (14)	11.3 (52)	0.12
State	23.5 (124)	23.5 (16)	23.5 (108)	
Local	61.7 (326)	1.5 (1)	63.3 (291)	
Non-profit	1.7 (9)	1.5 (1)	1.5 (7)	
Other	0.6 (3)	2.9 (2)	0.4 (2)	

^a^Multiple responses were possible.

### Procedure

Participants were recruited through a variety of means connected with the National Criminal Justice Training Center (NCJTC). Specifically, recruitment occurred through announcements at the July 2021 ICAC Virtual Conference, the October 2021 ICAC Virtual Commanders Meeting, during NCJTC trainings, through the ICAC listserv, and through specific invitations to past NCJTC students with “forensic” in their title.

Participants completed an anonymous survey hosted through Qualtrics, and online survey data collection system. Participants were told the aim of the study was to understand the impact of work-related exposure to CSAM. The data collection period was July 2021 December 2021. Participants were told they could skip any questions they did not want to answer. To ensure full anonymity, we turned off all Qualtrics tracking features, like IP address, longitude or latitude. We also encouraged participants to take the survey while in “incognito” mode and instructed them on how to do this. The recruitment methodology using announcements at national conferences and trainings results in a convenience sample, in contrast to a probability sample, so a meaningful response rate cannot be calculated. At the end of the survey participants were provided with resources where they could learn more about trauma and wellbeing and to seek help if needed (e.g., National Suicide Prevention Lifeline, National Mental Health Information Center, the IACP mental wellness for police officers’ website). All data are collected under the approval of the [*masked for review*] Institutional Review Board.

### Measures

The measures consisted of a combination of established scales and those developed for the current study. Newly developed items were designed through interviews and consultations with criminal justice personnel and mental health providers.

*Child Sexual Abuse Material Exposures* was measured by 11 content exposure items which were combined to create a total content CSAM score (*α* = 0.95; *M* = 33.9, *SD* = 8.3). Specifically, participants were asked to indicate, “In a typical month, approximately how often do you review CSAM images or videos that… (1) include children age 5 or young, (2) include children age 6–10, (3) were graphic (focused on genitals or showed explicit activity), (4) involve penetration of a child, including oral sex, (5) involve violence, beyond the sexual assault, (6) involve nudity or semi-nudity, without being graphic, (6) involve suggested poses of minors with clothes on, (7) involve multiple children at the same time, (8) involve children clearly under the influence of drugs or alcohol, (9) involve multiple offenders, (10) involve fetishes (animals, costumes, role-playing, bondage), and (11) involve sound. Response options for each were never, sometimes, often, and all the time.

*Depression and anxiety* were measured using the Patient Health Questionnaire-4 (PHQ-4) (39). The scale presents a list of four problems, two about anxiety (e.g., “Feeling nervous, anxious or on edge”) and two about depression (e.g., “Feeling down, depressed or hopeless”). Participants were asked to indicate how much each problem had bothered them in the past 2 weeks from 0 (“not at all”) to 3 (“nearly every day”). Items were combined to create a total scale score (*α* = 0.84) with higher scores representing more symptomatology (*M* = 5.77, *SD* = 2.33).

*Posttraumatic stress disorder* was measured using the Posttraumatic Stress Disorder Checklist for DSM-5 (PCL-5) (40). The PCL-5 presents four reactions that some people have in response to a very stressful experience (e.g., feeling distant or cut off from other people) and asks respondents to indicate how much they have been bothered by each in the past month. Response options ranged from 1 (not at all) to 5 (extremely). Items were combined to create a total scale score (*α* = 0.79) with higher scores representing more PTSD symptomatology (*M* = 6.52, *SD* = 2.75).

*Intimate relationship satisfaction* was assessed using the Relationship Assessment Scale (RAS) (41) is a 7-item scale designed to measure general relationship satisfaction with a spouse or dating partner. Questions address a variety of relationship dynamics including overall satisfaction (e.g., in general, how satisfied are you with your relationship?) and emotional intimacy (e.g., “How much do you love your partner?”). Respondents answer each item using a 5-point scale ranging from 1 (low satisfaction) to 5 (high satisfaction). Items are summed to a continuous score with higher scores (*α* = 0.91) reflecting more satisfaction with his/her relationship (*M* = 25.55, *SD* = 4.90).

*Burnout* was assessed using a scale specifically developed for the current study. The scale was crafted in consultation with criminal justice personnel and mental health providers to align with the unique demands of the field. It consisted of 19 items, which probed how frequently participants endorsed a range of 19 feelings related to their work, encompassing both positive (e.g., useful, honored) and negative (e.g., hopeless, angry) emotions. For the purposes of our analyses, negative work attitudes were included as an indicator of burnout, comprising 12 items (*α* = 0.89). Respondents rated their experiences on a 5-point scale, ranging from 1 (never) to 5 (always). This scale, while inspired by existing measures (42), was tailored to the specific context of our study to comprehensively capture the nuances of burnout in this specific context.

*Group connectedness* was measured using a scale of six items measured connections with groups or teams (29). Respondents were provided the following introduction: “People belong to all sorts of groups. You may play sports or music. You might play on a hockey or baseball team, or belong to a hunting club, or another group with interests like your own. For the next questions, think about the groups or teams you have belonged to.” Sample items include “I belong to a group or team that… (a) means a lot to me, (b) where I learned about working together.” Response options ranged from 1 (not true about me) to 4 (mostly true about me). Items were combined to create a total scale score (*α* = 0.94) with higher scores representing more group connectedness.

*Social support seeking* was measured using six items that asked questions about friends and people who are special in their lives (29). Respondents were asked to think about the people you know in person and respond to questions about those people. Sample items include “Talking to someone who has been through the same thing helps me” and” I talk to someone to help me solve problems.” Response options ranged from 1 (not true about me) to 4 (mostly true about me). Items were combined to create a total scale score (*α* = 0.91) with higher scores representing more social support seeking.

*Participant demographics* included information about the respondents current job description, the types of crimes they investigate, years in current position and in law enforcement, whether they work as part of the Internet Crimes Against Children (ICAC) Task Force program, gender, age, race, ethnicity, marital status, number of children and/or grandchildren who are currently minors, and type of community (large city, small town, etc.).

### Data analysis

Data analysis consisted of a combination of bivariate statistics and multivariate statistics using StataSE 17.0. First, sample demographic characteristics were compared between participants who said they were and were not in a relationship at the time of the study. Then, bivariate pairwise correlations were conducted between the main study constructs. Descriptive statistics were also analyzed for these constructs. Finally, three liner regression analyses were conducted to examine the relationships between mental health and resiliency (i.e., group connectedness and social support seeking) and intimate relationship satisfaction among: (1) all participants, (2) male participants, and (3) female participants.

## Results

### Descriptive statistics and correlations

Means and standard deviations of all main study constructs are detailed in Table 2. No significant mean score differences were noted between male and female participants for intimate relationship satisfaction. The only differences based on gender were scores on anxiety and social support seeking with females having higher mean scores on both.

**Table 2:** Descriptive statistics for main study constructs by gender.

Construct	*N*	All (*n* = 460) *M* (*SD*)	Male (*n* = 295) *M* (*SD*)	Female (*n* = 165) *M* (*SD*)	t statistic	*p-*value
Relationship satisfaction	460	25.6 (4.9)	25.6 (4.8)	25.5 (5.1)	0.17	0.86
Depression	527	1.2 (1.4)	1.3 (1,5)	1.2 (1.4)	0.91	0.36
Anxiety	523	1.7 (1.6)	1.5 (1.6)	2.0 (1.7)	−2.77	0.006
PTSD	528	6.5 (2.7)	6.5 (2.7)	6.6 (2.8)	−0.30	0.76
Burnout	512	30.0 (7.1)	30.1 (7.2)	29.9 (6.9)	0.26	0.79
Group connectedness	528	14.5 (5.2)	14.5 (5.2)	14.6 (5.4)	−0.27	0.78
Social support seeking	528	15.3 (3.9)	14.8 (3.9)	16.1 (3.9)	−3.72	<0.001

Pairwise correlations indicated that all the main study constructs were significantly correlated with one another with the exception of CSAM content exposure which was not significantly related to any other construct and thus not included in further analyses (see Table 3). Similarly, alcohol use was not related to intimate relationship satisfaction and thus was excluded from the final regression models. Intimate relationship satisfaction was also significantly positively correlated with group connectedness (*r* = 0.21, *p* < 0.001), social support seeking (*r* = 0.13, *p* < 0.01), and purpose in life (*r* = 0.30, *p* < 0.001) and significantly negatively correlated with depression (*r* = −0.22, *p* < 0.001), anxiety (*r* = −0.12, *p* < 0.01), PTSD (*r* = −0.20, *p* < 0.001), and burnout (*r* = −0.10, *p* < 0.05).

**Table 3:** Correlations among key study constructs.

		1	2	3	4	5	6	7	8
1	Intimate relationship satisfaction	1.0							
2	Depression	−0.22***	1.0						
3	Anxiety	−0.12**	0.60***	1.0					
4	PTSD	−0.20***	0.55***	0.45***	1.0				
5	Burnout	−0.10*	0.56***	0.48***	0.50***	1.0			
6	CSAM exposure	−0.01	0.001	−0.01	0.03	0.07	1.0		
7	Group connectedness	0.21***	−0.27***	−0.13**	−0.19***	−0.25***	−0.03	1.0	
8	Social support seeking	0.13**	−0.24***	−0.07	−0.14**	−0.16***	−0.08	0.29***	1.0

*p < 0.05, **p < 0.01, ***p < 0.001.

### Relationships between mental health, resiliency, and intimate relationship satisfaction

Participants with higher levels of depressive symptoms (*β* = −0.16, *p* = 0.01) and PTSD symptoms (*β* = −0.12, *p* = 0.03) scored significantly lower on intimate relationship satisfaction (Table 4). Participants who were parents also scored lower on intimate relationship satisfaction (*β* = −0.11, *p* = 0.02). Intimate relationship satisfaction was higher for those who scored higher on ground connectedness (*β* = 0.16, *p* = 0.001). When this model was run separately for men and women, PTSD (*β* = −0.24, *p* = 0.001) and being a parent (*β* = −0.21, *p* < 0.001) were related to lower intimate relationship satisfaction for men while depression was related to lower intimate relationship satisfaction (*β* = −0.21, *p* = 0.04) for women. For both men (*β* = 0.13, *p* = 0.04) and women (*β* = 0.21, *p* = 0.01), group connectedness was related to more satisfaction.

**Table 4:** Multivariate linear regression of constructs related to intimate relationship satisfaction—overall and for men and women.

Construct	All participants (*n* = 441)		Men (*n* = 283)		Women (*n* = 158)	
	*β*	*P-*value	*β*	*P-*value	*β*	*P*-value
Depression	−0.16	0.01	−0.10	0.25	−0.21	0.04
Anxiety	0.02	0.74	0.06	0.43	−0.03	0.74
PTSD	−0.12	0.03	−0.24	0.001	0.07	0.43
Burnout	0.11	0.07	0.14	0.06	0.02	0.81
Group connectedness	0.16	0.001	0.13	0.04	0.21	0.01
Social support seeking	0.05	0.33	0.08	0.18	−0.01	0.92
Married	0.07	0.12	0.04	0.46	0.11	0.17
Parent	−0.11	0.02	−0.21	<0.001	0.06	0.45
Female	−0.03	0.54	–	–	–	–

## Discussion

The findings from this study shed light on the relationships between mental health, resiliency, and intimate relationship satisfaction among investigators of CSAM. Consistent with the initial hypotheses, results revealed that higher levels of depressive symptoms and PTSD were associated with lower intimate relationship satisfaction, indicating that investigators experiencing these mental health challenges may have less satisfying intimate relationships. Burnout was not found to be significantly associated with intimate relationship satisfaction in the multivariate model, but was highly correlated with mental health challenges. Similarly, the hypothesis that group connectedness would be associated with higher intimate relationship satisfaction was also supported.

Findings regarding mental health’s negative relationship to intimate relationship satisfaction align with previous research conducted among other populations exposed to trauma. Similar associations have been observed in studies involving military members, veterans, first responders, and survivors of sexual assault or childhood maltreatment (14, 16–19). Notably, burnout was significantly related to intimate relationship satisfaction in the bivariate model, but was not significant in the multivariate model. This may be due to the relationship between burnout and mental health, which is mirrored in prior studies (25, 43). Importantly, in the current study, PTSD specifically was related to intimate relationship dissatisfaction for men, while depression was significant for women. It is possible these gender differences might be related to the varying coping strategies men and women investigators may employ when dealing with the psychological impact of their work. Women investigators may be more inclined to internalize their distress, leading to depressive symptoms, while men investigators may be more likely to exhibit symptoms of PTSD as they attempt to cope with the traumatic experiences externally. Societal expectations and stigmas surrounding gender roles and mental health (44) may also possibly influence the reporting and interpretation of symptoms. Women may be more comfortable expressing depressive symptoms due to greater societal acceptance and awareness of depression in women, while men may be more reluctant to acknowledge and report depressive symptoms due to societal expectations of emotional strength (45, 46), resulting in an underreporting of depression and a heightened emphasis on PTSD symptoms. These findings suggest that the adverse psychological consequences of trauma exposure can extend beyond the individual and impact their intimate relationships (7). Although we did not find an overall relationship between content exposure and relationship satisfaction, it is important to acknowledge that the challenges and stressors inherent in CSAM investigation work can still take a toll on the mental health of investigators. Repeated exposure to graphic content can have significant psychological impacts, which can indirectly affect their ability to maintain satisfying and fulfilling intimate relationships. Moreover, the findings of this study support the applicability of the Spillover Model (15, 21, 23) in the context of CSAM investigations and highlight the importance of considering the broader impact of stressors in CSAM investigator’s work, on their intimate relationships.

Interestingly, being a parent was also found to be associated with lower relationship satisfaction for men but not for women. Naturally, challenges associated with parenthood can contribute to heightened stress and strain in relationships (47). However, within the context of CSAM investigations, where individuals are exposed to graphic content of child sexual abuse, the challenges faced by parent investigators may be even more pronounced. The constant exposure to such distressing material can result in increased projection of worries and anxiety onto their own children, potentially affecting their ability to maintain satisfying relationships (7). Therefore, it is important to acknowledge the unique challenges faced by investigators in the CSAM field who are also parents and provide them with tailored support systems and resources. The finding that parenthood is associated with lower relationship satisfaction for men but not for women can be understood by different factors. First, men and women often have different ways of dealing with stress, and societal norms may place different pressures on them in their roles as parents and protectors. While this general trend may hold, individual differences can also significantly influence how someone responds to the challenges of investigating CSAM. Moreover, the constant exposure to distressing material in this field can lead to increased worries and anxiety, potentially affecting men more profoundly in their traditional role as protectors. Recognizing these differences is crucial, and providing tailored support systems and resources for parent investigators in the CSAM field can help them better navigate these challenges and maintain satisfying relationships.

Of note, group connectedness was significantly associated with intimate relationship satisfaction for both men and women. The positive association between group connectedness and relationship satisfaction highlights the importance of broader interpersonal connections in fostering healthy and fulfilling relationships (7). Being part of a supportive group can provide investigators with a sense of belonging, understanding, and validation, which can contribute to enhanced relationship satisfaction. Further, it may be that connection to a positive social group aids in reminding investigators of positive communities and relationships wherein abuse and violence are not occurring. Previous research with professionals exposed to trauma suggests that compartmentalizing personal and work lives can be protective against ongoing stress and vicarious trauma (48). Accordingly, it may be that CSAM investigators connected with a group are better able to effectively leave their work “at work” while engaging in an extracurricular interest (authors own, blinded).

The findings showed a lack of support for the hypothesis that social support seeking would have a positive relationship to intimate relationship satisfaction. This result was somewhat surprising and converse to previous findings [e.g., (49)]. It may be that CSAM investigators have a different experience of social support seeking, given their jobs and unique responsibilities. For example, social support seeking is assessed through questions such as “talking things out when one is upset.” However, if an investigator is upset about a case they are working on or content they see, talking things out may be discouraged, unsupported, or even inappropriate in the context of the larger CSAM investigation. Furthermore, given the previous finding that CSAM investigators prefer to “shut off” after work (7), along with the findings that varied professionals prefer to compartmentalize their home and work lives separately (authors own, blinded), it may be that talking with a partner less is indicative of better coping and a healthier intimate relationship. It is important to note that the survey was looking exclusively at investigators’ opinions and did not gather information from their partners’ perspectives regarding support seeking or intimate relationship satisfaction. It may be that partners of those investigators who report low social support seeking and high relationship satisfaction feel differently than their partner about one or both of these constructs.

Finally, it is notable that CSAM content exposure was not significantly related to the other constructs measured. It is unclear why this may be. It may be that those with successful coping strategies remain resilient regardless of their exposure, while investigators with less successful coping, struggle regardless of the exposure or severity of CSAM exposure. Indeed, previous research has championed the ubiquity of positive interpersonal connection in protecting individuals from a variety of adverse effects (32, 50). Given the vast diversity of CSAM exposure among the current sample, it is likely that there is some universality in experience for investigators around CSAM exposure and that some unknown construct changes its direct impact on outcomes.

### Limitations and future research directions

The current study had a few limitations that should be noted when considering the implications of the findings. First, data were collected via a convenience sample, which might not be representative of the population of investigators/forensic examiners who view CSAM. Moreover, it is possible, that there was a built-in bias to a study on police wellness, in which law enforcement who are more resilient—or more troubled—were the ones more willing to complete a survey on their CSAM exposure and mental health and wellbeing. Second, the study was based on self-report measures, which are subject to response bias (e.g., under- or over-reporting). Investigators may have particular biases against acknowledging mental health symptoms (51). Third, the design was cross-sectional; therefore, causal relationships between mental health and intimate relationship satisfaction cannot be drawn. While our findings suggest that mental health issues are negatively associated with intimate relationship satisfaction, it is important to recognize the bidirectional nature of this relationship. Difficulties within intimate relationships can also contribute to or intensify existing mental health challenges. Therefore, it is crucial to consider the reciprocal influence between mental health and relationship satisfaction when developing interventions and support strategies. Finally, it is important to note that despite high exposure rates to CSAM, mental health and PTSD levels are within the normal range or at a mild level. This finding calls for a deeper examination and suggests the possibility of resilience within the sample. However, it also raises questions about the complex relationship between exposure and mental health outcomes. To better understand these dynamics, further investigation is necessary to explore potential moderating variables that could influence the connection between exposure and mental health outcomes.

### Implications

Despite these limitations, the current study offers several important implications for research and practice.

#### Research

The current study highlights the experiences of investigators exposed to CSAM, and it would be useful to understand more about how these results may be replicated- or not- among investigators of other crimes such as homicide, theft, or missing/exploited youth. It would be interesting to understand the unique impact of CSAM content on investigators so that supports could be tailored to fit investigator needs, thereby maximizing effectiveness and minimizing burnout. Furthermore, it would be important for future research to consider the long-term health of officer relationships and connections, including data triangulation from the families/partners of investigators. A longitudinal approach would be valuable in exploring the dynamic interactions between mental health and relationship satisfaction among CSAM investigators over time. Finally, future research should consider employing dyadic data collection methods to measure secondary trauma and satisfaction of both the CSAM investigators and their partners. This would provide a comprehensive understanding of the relationship dynamics and experiences of both individuals. Such dynamic and longitudinal data would undoubtedly shed light on long-term trends and trajectories that could be halted and redirected with pointed intervention.

#### Clinical

The current study highlights the many nuances experiences of investigators who are exposed to CSAM, and underscores the importance of holistic wellbeing. Law enforcement agencies must find new and innovative ways to encourage officers to engage in meaningful relationships, both in and outside the law enforcement agency. Given the findings of the current study, encouraging CSAM investigators to engage in group activities both in and out of the office may be an important first step. Similarly, fostering agency environments wherein mental health is neither stigmatized nor ignored could ultimately impact other areas of investigator’s lives, including their intimate relationships. Law enforcement agencies might consider interventions that address both mental health concerns and relationship dynamics simultaneously, aiming for a more comprehensive and effective approach to support the wellbeing of individuals in their relationships.

## Conclusion

The current study offers an important initial glance into the lives of CSAM investigators correlates of healthy (and less healthy) intimate relationships. Given the emotional toll their work takes, as well as the importance of preventing investigator burnout, it is important that we continue to find new and innovative ways to foster the wellbeing and intimate relationships of investigators who are exposed to CSAM.

## Data availability statement

The datasets presented in this article are not readily available because data is confidential. Requests to access the datasets should be directed to Kimberly.Mitchell@unh.edu.

## Ethics statement

The studies involving humans were approved by the University of New Hampshire IRB (IRB-FY2022-104). The studies were conducted in accordance with the local legislation and institutional requirements. The participants provided their written informed consent to participate in this study.

## Author contributions

AG-M, JO’B, and KM: conceptualization, interpretation of results, writing—review and editing. KM: methodology development, data collection, statistical analysis, funding acquisition, project administration. AG-M: writing—original draft preparation. All authors contributed to the article and approved the submitted version.
